# Supplementary material for: Alteration of Fatty Acid Profile in Fragile X Syndrome
Source: Int J Mol Sci. 2022 Sep 16;23(18):10815. doi: 10.3390/ijms231810815 (PMC9502195; doi:10.3390/ijms231810815)
Supplement: Supplementary file 1 [file ijms-23-10815-s001.zip › ijms-1832985-supplementary.pdf]

Table S1. FA profile in male group

Fatty acids	Fragile X ($n = 21$) Median [25% and 75% percentile]	Healthy Control ($n = 21$) Median [25% and 75% percentile]	*Adjusted P-Value*
C14:0	7.8 [6.7, 9.1]	9.6 [7.4, 11.00]	***0.0213***
C16:0	488.2 [444.8, 529.1]	592.2 [536.6, 684.1]	***0.0006***
C18:0	362.3 [274.7, 396.9]	382.4 [326.4, 461.1]	*0.1038*
SFA total	849.3 [745.1, 957.5]	975.1 [883.0, 1094.0]	***0.0011***
C16:1 ω-7	6.4 [5.6, 8.3]	9.9 [8.1, 14.0]	***0.0006***
C18:1 ω-9	205.6 [181.9, 239.2]	240.7 [216.2, 253.3]	***0.0137***
C18:1 ω-7	20.8 [16.8, 26.0]	26.7 [20.4, 31.2]	***0.0058***
MUFA total	232.5 [202.2, 272.8]	272.8 [245.0, 300.5]	***0.0084***
C18:2 ω-6	371.4 [308.3, 433.6]	456.8 [391.7, 522.9]	***0.0006***
C20:3 ω-6	55.0 [38.5, 75.6]	65.5 [55.1, 86.6]	***0.0362***
C20:4 ω-6	182.6 [152.0, 255.7]	213.9 [173.6, 282.6]	***0.0446***
ω-6 total	621.3 [527.8, 716.7]	757.2 [650.1, 868.4]	***0.0009***
C18:3 ω-3	2.8 [2.3, 3.8]	3.8 [2.4, 4.3]	*0.1055*
C20:5 ω-3	6.5 [4.4, 10.0]	12.5 [6.9, 15.4]	***0.0037***
C22:5 ω-3	15.6 [11.5, 19.4]	17.1 [15.4, 22.5]	*0.1298*
C22:6 ω-3	43.2 [34.4, 55.0]	58.1 [37.4, 80.8]	***0.0289***
ω-3 total	70.8 [57.1, 85.0]	93.3 [68.2, 127.0]	***0.0137***
PUFA total	679.2 [600.6, 799.6]	842.4 [727.0, 969.0]	***0.0010***
ω-3 / ω-6	0.1 [0.1, 0.1]	0.1 [0.1, 0.1]	*0.3526*
C20:5/C18:3(EPA/ALA)	2.4 [1.6, 3.7]	3.1 [2.7, 4.0]	*0.0505*
C22:5/C20:5(DPA/EPA)	2.5 [1.4, 3.2]	1.5 [1.2, 2.2]	***0.0336***
C22:6/C22:5(DHA/DPA)	2.7 [2.2, 3.3]	3.3 [2.3, 4.2]	*0.1590*
C22:6/C20:5(DHA/EPA)	6.6 [3.6, 10.2]	5.3 [2.7, 7.4]	*0.1298*
20:3/18:2	0.2 [0.1, 0.2]	0.1 [0.1, 0.2]	*0.5916*
20:4/20:3	3.8 [2.6, 4.5]	3.3 [2.6, 4.5]	*0.4474*

Table S2. FA profile in adult cohort

**Fatty acids**	**Fragile X (*n* = 21) Median [25% and 75% percentile]**	**Healthy Control (*n* = 21) Median [25% and 75% percentile]**	***Adjusted P-Value***
C14:0	8.1 [6.3, 8.8]	9.5 [7.2, 10.7]	*0.1012*
C16:0	466.9 [414.8, 547.2]	619.7 [574.9, 684.1]	***0.0004***
C18:0	312.9 [250.4, 389.3]	382.4 [301.8, 461.1]	***0.0492***
SFA total	797.1 [680.3, 972.8]	1041.0 [903.5, 1147.0]	***0.0029***
C16:1 ω-7	5.9 [5.6, 7.7]	11.6 [8.3, 14.8]	***0.0057***
C18:1 ω-9	189.7 [152.1, 233.8]	240.7 [216.2, 256.4]	***0.0153***
C18:1 ω-7	22.4 [17.6, 27.1]	28.5 [24.0, 32.3]	***0.0032***
MUFA total	221.1 [178.6, 268.4]	274.7 [261.2, 298.6]	***0.0104***
C18:2 ω-6	324.0 [279.4, 406.1]	500.6 [445.2, 532.0]	***0.0004***
C20:3 ω-6	53.6 [38.2, 74.0]	67.8 [55.3, 84.8]	*0.0586*
C20:4 ω-6	175.3 [124.8, 238.3]	251.0 [177.1, 322.1]	***0.0082***
ω-6 total	549.8 [476.6, 696.9]	768.8 [709.5, 912.8]	***0.0004***
C18:3 ω-3	3.0 [2.2, 4.8]	4.1 [2.9, 6.6]	*0.0942*
C20:5 ω-3	7.1 [6.0, 13.0]	14.3 [12.3, 16.3]	***0.0057***
C22:5 ω-3	16.8 [11.4, 19.7]	18.8 [16.6, 24.1]	*0.138*
C22:6 ω-3	44.1 [32.1, 57.7]	67.8 [37.9, 81.4]	***0.0143***
ω-3 total	74.2 [55.1, 100.8]	104.1 [85.2, 134.0]	***0.0082***
PUFA total	608.3 [542.0, 803.5]	872.8 [811.1, 1074.0]	***0.0004***
ω-3 / ω-6	0.1 [0.1, 0.2]	0.1 [0.1, 0.1]	*0.5893*
C20:5/C18:3(EPA/ALA)	2.9 [1.9, 4.5]	3.6 [3.0, 4.2]	*0.1472*
C22:5/C20:5(DPA/EPA)	1.5 [1.1, 2.7]	1.4 [1.1, 1.6]	*0.138*
C22:6/C22:5(DHA/DPA)	2.8 [2.4, 3.2]	3.6 [2.4, 4.3]	*0.1568*
C22:6/C20:5(DHA/EPA)	3.2 [2.8, 5.6]	4.3 [3.6, 5.3]	
20:3/18:2	0.2 [0.1, 0.2]	0.1 [0.1, 0.2]	*0.1963*
20:4/20:3	3.2 [2.5, 4.5]	3.5 [2.7, 4.9]	*0.3381*

Table S3. FA profile in children cohort

Fatty acids	Fragile X ($n = 9$) Median [25% and 75% percentile]	Healthy Control ($n = 9$) Median [25% and 75% percentile]	*Adjusted P-Value*
C14:0	9.1 [7.2, 9.3]	9.7 [8.9, 11.7]	*0.7903*
C16:0	501.9 [472.3, 537.4]	536.6 [503.,4, 561.2]	*0.8341*
C18:0	380.0 [353.4, 456.7]	373.3 [340.8, 401.3]	*0.8612*
SFA total	911.7 [834.5, 980.4]	904.4 [866.0, 972.8]	*0.9785*
C16:1 ω-7	6.7 [5.6, 10.1]	9.2 [7.4, 11.2]	*0.7903*
C18:1 ω-9	220.2 [184.9, 279.1]	245.8 [207.5, 260.9]	*0.8612*
C18:1 ω-7	17.0 [15.2, 21.6]	18.8 [16.2, 24.4]	*0.8612*
MUFA total	249.8 [205.6, 307.1]	274.1 [233.0, 294.2]	*0.8612*
C18:2 ω-6	421.2 [359.8, 497.7]	391.7 [359.7, 436.5]	*0.8612*
C20:3 ω-6	56.92 [45.7, 79.0]	67.44 [45.9, 85.1]	*0.8612*
C20:4 ω-6	193.4 [173.0, 222.8]	182.2 [139.8, 245.2]	*1*
ω-6 total	704.3 [588.3, 778.0]	650.1 [568.1, 729.4]	*0.9908*
C18:3 ω-3	2.6 [1.9, 3.4]	2.4 [2.2, 3.0]	*0.9785*
C20:5 ω-3	4.39 [4.3, 7.2]	7.2 [4.4, 13.3]	*0.8341*
C22:5 ω-3	15.8 [12.2, 19.2]	16.2 [11.7, 19.2]	*0.9785*
C22:6 ω-3	42.1 [32.9, 55.6]	45.8 [31.9, 62.2]	*1*
ω-3 total	72.1 [56.0, 76.1]	69.0 [59.7, 88.2]	*0.9785*
PUFA total	776.4 [666.3, 841.2]	727.0 [628.3, 810.9]	*0.8983*
ω-3 / ω-6	0.1 [0.1,0.1]	0.1 [0.1, 0.1]	*0.8341*
C20:5/C18:3(EPA/ALA)	2.2 [1.6, 2.9]	3.1 [2.4, 4.7]	*0.7903*
C22:5/C20:5(DPA/EPA)	2.5 [2.2, 4.2]	2.5 [1.5, 3.0]	*0.8341*
C22:6/C22:5(DHA/DPA)	2.5 [2.1, 4.4]	3.1 [1.9, 4.1]	*1*
C22:6/C20:5(DHA/EPA)	10.0 [5.5, 13.3]	6.4 [5.1, 9.4]	*0.8341*
20:3/18:2	0.1 [0.1, 0.2]	0.2 [0.1, 0.2]	*0.8612*
20:4/20:3	3.7 [2.6, 4.2]	3.0 [2.4, 3.5]	*0.8341*

Table S4. FA profile in adolescence cohort

Fatty acids	Fragile X (n = 4) Median [25% and 75% percentile]	Healthy Control (n = 4) Median [25% and 75% percentile]	*Adjusted P-Value*
C14:0	7.4 [7.2, 9.3]	8.3 [6.0, 10.9]	*0.9723*
C16:0	495.6 [454.4, 520.5]	560.5 [518.4, 596.1]	*0.5611*
C18:0	386.3 [344.1, 451.2]	451.9 [446.7, 469.2]	*0.5611*
SFA total	890.7 [805.9, 979.5]	1031.0 [978.4, 1059.0]	*0.5611*
C16:1 ω-7	8.0 [5.8, 8.4]	8.7 [8.3, 10.0]	*0.5611*
C18:1 ω-9	229.8 [175.4, 264.2]	245.3 [236.8, 286.8]	*0.5611*
C18:1 ω-7	20.8 [17.2, 25.0]	22.2 [20.6, 29.0]	*0.6813*
MUFA total	258.9 [199.1, 278.5]	275.1 [267.2, 325.2]	*0.5611*
C18:2 ω-6	395.9 [362.9,445.4]	475.5 [411.9, 507.7]	*0.5611*
C20:3 ω-6	53.38 [31.9, 71.7]	63.3 [60.7, 82.8]	*0.5611*
C20:4 ω-6	181.8 [155.2, 290.0]	205.2 [185.4, 209.9]	*0.6813*
ω-6 total	616.3 [565.4, 806.4]	743.1 [676.8, 782.5]	*0.6813*
C18:3 ω-3	2.3 [2.0, 3.4]	2.4 [2.3, 3.9]	*0.6813*
C20:5 ω-3	3.5 [3.0, 7.9]	5.9 [4.0, 6.6]	*0.6813*
C22:5 ω-3	12.1 [9.9, 21.4]	14.7 [11.4, 16.1]	*0.787*
C22:6 ω-3	45.7 [38.4, 57.3]	51.0 [44.1, 70.2]	*0.6813*
ω-3 total	68.5 [55.8, 82.4]	74.3 [63.1, 95.3]	*0.787*
PUFA total	684.9 [621.3, 888.8]	812.6 [744.7, 877.8]	*0.6813*
ω-3 / ω-6	0.1 [0.1, 0.1]	0.1 [0.1, 0.1]	*1*
C20:5/C18:3(EPA/ALA)	1.6 [1.4, 2.3]	1.9 [1.4, 2.8]	*1*
C22:5/C20:5(DPA/EPA)	3.2 [2.8, 3.6]	2.5 [2.0, 3.7]	*0.6813*
C22:6/C22:5(DHA/DPA)	3.3 [2.3, 5.8]	3.8 [3.3, 4.7]	*0.6813*
C22:6/C20:5(DHA/EPA)	11.6 [6.7, 18.6]	9.8 [7.6, 13.7]	*0.787*
20:3/18:2	0.1 [0.1, 0.2]	0.1 [0.1, 0.2]	*0.787*
20:4/20:3	3.9 [3.0, 6.2]	3.1 [2.4, 3.5]	*0.5611*

Table S5. FA profile in Argentinian group

Fatty acids	Fragile X (n = 23) Median [25% and 75% percentile]	Healthy Control (n = 23) Median [25% and 75% percentile]	Adjusted *P-Value*
C14:0	8.5[7.1, 9.2]	9.8 [8.7, 11.1]	**0.0149**
C16:0	504.3 [456.5, 536.1]	572.0 [536.6, 625.5]	**0.0046**
C18:0	377.4 [356.4, 435.8]	406.3 [373.3, 463.5]	0.1646
SFA total	899.9 [826.3, 978.1]	982.1 [934.9, 1094.0]	**0.0149**
C16:1 ω-7	6.9 [5.6, 8.8]	9.9 [8.3, 11.6]	**0.0046**
C18:1 ω-9	215.9 [183.7, 248.7]	246.6 [234.8, 264.8]	**0.0194**
C18:1 ω-7	20.2 [16.6, 23.3]	22.3 [19.3, 27.7]	0.1296
MUFA total	242.3 [208.8, 282.6]	279.3 [265.3, 306.0]	**0.0209**
C18:2 ω-6	389.4 [326.5, 449.9]	451.7 [382.0, 490.9]	**0.0475**
C20:3 ω-6	57.6 [51.9, 77.2]	67.8 [59.3, 86.6]	0.1539
C20:4 ω-6	195.6 [172.9, 236.1]	213.9 [182.2, 271.9]	0.2949
ω-6 total	660.4 [556.9, 729.6]	733.4 [667.2, 783.1]	**0.0411**
C18:3 ω-3	2.4 [1.9, 3.3]	2.5 [2.3, 3.8]	0.1790
C20:5 ω-3	4.8 [4.0, 9.2]	9.8 [5.8, 14.3]	**0.0238**
C22:5 ω-3	15.6 [11.5, 19.4]	17.0 [14.0, 21.3]	0.4769
C22:6 ω-3	47.6 [36.8, 60.6]	58.1 [42.0. 70.0]	0.2075
ω-3 total	72.3 [57.6, 85.0]	93.3 [61.6, 104.1]	0.1689
PUFA total	732.4 [631.8, 815.9]	822.5 [737.5, 884.1]	**0.0475**
ω-3 / ω-6	0.1 [0.1, 0.1]	0.1 [0.1, 0.1]	0.4129
C20:5/C18:3(EPA/ALA)	2.3 [1.6, 3.1]	3.1 [2.7, 4.5]	**0.0361**
C22:5/C20:5(DPA/EPA)	2.8 [2.3, 3.7]	1.8 [1.4, 2.7]	**0.0149**
C22:6/C22:5(DHA/DPA)	2.8 [2.3, 4.0]	3.5 [2.4, 4.0]	0.3577
C22:6/C20:5(DHA/EPA)	8.4 [6.6, 11.4]	5.8 [4.8, 8.0]	**0.0473**
20:3/18:2	0.2 [0.1, 0.2]	0.2 [0.1, 0.2]	0.8783
20:4/20:3	3.7 [2.6, 4.4]	3.1 [2.6, 3.6]	0.2889

Table S6. FA profile in French Canadian group

**Fatty acids**	**Fragile X (n = 11) Median [25% and 75% percentile]**	**Healthy Control (n = 11) Median [25% and 75% percentile]**	***Adjusted P-Value***
C14:0	7.3 [6.1, 10.0]	7.4 [3.9, 10.4]	0.7698
C16:0	417.2 [351.1, 523.9]	592.2 [527.0, 684.1]	**0.0394**
C18:0	253.6 [229.5, 312.4]	301.8 [281.6, 352.8]	0.1341
SFA total	682.6 [604.7, 843.5]	903.5 [815.5, 1041.0]	0.0534
C16:1 ω-7	5.9 [5.6, 7.3]	14.0 [5.5, 14.9]	0.2454
C18:1 ω-9	182.2 [148.7, 225.1]	216.2 [150.3, 231.8]	0.4936
C18:1 ω-7	22.4 [16.7, 27.2]	30.3 [28.3, 34.2]	**0.0101**
MUFA total	212.9 [176.2, 260.2]	265.4 [186.0, 274.7]	0.2454
C18:2 ω-6	307.6 [271.4, 387.9]	500.6 [438.7, 522.9]	**0.0309**
C20:3 ω-6	38.9 [36.5, 64.3]	57.0 [44.2, 83.0]	0.2454
C20:4 ω-6	170.3 [95.32, 184.6]	180.6 [163.2, 332.9]	0.1341
ω-6 total	519.5 [430.8, 620.7]	757.2 [628.8, 912.8]	**0.0309**
C18:3 ω-3	4.1 [2.8, 5.9]	5.8 [4.1, 7.4]	0.2454
C20:5 ω-3	10.2 [6.6, 19.7]	14.5 [12.6, 33.7]	0.1425
C22:5 ω-3	14.3 [10.5, 19.8]	18.8 [15.9, 24.6]	0.2605
C22:6 ω-3	39.5 [25.3, 44.4]	80.8 [37.3, 98.8]	0.1341
ω-3 total	66.3 [44.0, 98.3]	133.9 [73.3, 142.9]	**0.0505**
PUFA total	593.7 [474.7, 698.3]	842.4 [702.1, 1074.0]	**0.0309**
ω-3 / ω-6	0.1 [0.1, 0.2]	0.1 [0.1, 0.2]	0.8779
C20:5/C18:3(EPA/ALA)	2.6 [1.4, 4.4]	3.0 [2.2, 3.7]	0.8779
C22:5/C20:5(DPA/EPA)	1.2 [1.0, 1.9]	1.2 [1.1, 1.5]	0.9452
C22:6/C22:5(DHA/DPA)	2.6 [2.2, 2.9]	2.5 [2.3, 5.3]	0.4936
C22:6/C20:5(DHA/EPA)	3.5 [2.8, 4.7]	2.7 [2.4, 8.1]	0.8779
20:3/18:2	0.2 [0.1, 0.2]	0.1 [0.1, 0.2]	0.4936
20:4/20:3	3.1 [2.5, 4.5]	3.8 [2.7, 5.8]	0.4936
